# Disruption in proprioception from long-term thalamic deep brain stimulation: a pilot study

**DOI:** 10.3389/fnhum.2015.00244

**Published:** 2015-05-01

**Authors:** Jennifer A. Semrau, Troy M. Herter, Zelma H. Kiss, Sean P. Dukelow

**Affiliations:** ^1^Hotchkiss Brain Institute, University of CalgaryCalgary, AB, Canada; ^2^Department of Clinical Neurosciences, University of CalgaryCalgary, AB, Canada; ^3^Department of Exercise Science, University of South CarolinaColumbia, SC, USA

**Keywords:** proprioception, tremor, deep brain stimulation (DBS), kinesthesia, position sense, sensorimotor

## Abstract

Deep brain stimulation (DBS) is an excellent treatment for tremor and is generally thought to be reversible by turning off stimulation. For tremor, DBS is implanted in the ventrointermedius (Vim) nucleus of the thalamus, a region that relays proprioceptive information for movement sensation (kinaesthesia). Gait disturbances have been observed with bilateral Vim DBS, but the long-term effects on proprioceptive processing are unknown. We aimed to determine whether Vim DBS surgical implantation or stimulation leads to proprioceptive deficits in the upper limb. We assessed two groups of tremor subjects on measures of proprioception (kinaesthesia, position sense) and motor function using a robotic exoskeleton. In the first group (Surgery), we tested patients before and after implantation of Vim DBS, but before DBS was turned on to determine if proprioceptive deficits were inherent to tremor or caused by DBS implantation. In the second group (Stim), we tested subjects with chronically implanted Vim DBS ON and OFF stimulation. Compared to controls, there were no proprioceptive deficits before or after DBS implantation in the Surgery group. Surprisingly, those that received chronic long-term stimulation (LT-stim, 3–10 years) displayed significant proprioceptive deficits ON and OFF stimulation not present in subjects with chronic short-term stimulation (ST-stim, 0.5–2 years). LT-stim had significantly larger variability and reduced workspace area during the position sense assessment. During the kinesthetic assessment, LT-stim made significantly larger directional errors and consistently underestimated the speed of the robot, despite generating normal movement speeds during motor assessment. Chronic long-term Vim DBS may potentially disrupt proprioceptive processing, possibly inducing irreversible plasticity in the Vim nucleus and/or its network connections. Our findings in the upper limb may help explain some of the gait disturbances seen by others following Vim DBS.

## Introduction

The human ventrointermedius nucleus (Vim) of thalamus is comprised of neurons that respond to sensation of movement, or kinaesthesia (Hirai et al., 1989; Ohye et al., 1989). While Vim is the most common surgical target for deep brain stimulation (DBS) to relieve tremor, the underlying mechanisms responsible for its effectiveness are unknown. Current work suggests that Vim DBS suppresses tremor by disrupting rhythmic cell firing, either by neurotransmitter depletion (Anderson et al., 2004, 2006) or additional filling in of cellular firing, essentially blocking the rhythmic firing of tremor cells (Kiss et al., 2002; Grill et al., 2004). It is thought that DBS “normalizes” subcortical firing transmitted to sensorimotor cortices, resulting in suppressed tremor and more normal motor output (Perlmutter and Mink, 2006).

The mapping of human Vim during surgery has provided useful information concerning the functional organization of kinaesthetic behavior (Hirai et al., 1989; Ohye et al., 1989). Kinaesthesia, or sense of movement, is one of two sensory submodalities traditionally thought to comprise proprioception, the other being position sense (Sherrington, 1907). Proprioception is necessary for the proper generation and guidance of movement (Todorov and Jordan, 2002). From peripheral receptors, kinaesthetic information (sensation of direction, magnitude, and speed) is transmitted via the dorsal column and spinocerebellar pathways to the Vim nucleus and then to somatosensory and motor cortices (Mountcastle, 1958; Hassler, 1959; Narabayashi, 1989).

Vim DBS is safe and extremely effective for long-term suppression of tremor in patients with essential tremor (ET) or Parkinson (PD) tremor (Benabid et al., 1991; Hubble et al., 1996; Schuurman et al., 2000; Pahwa et al., 2006). DBS started as an alternative to thalamotomy, or surgical lesioning of Vim (Benabid et al., 1996). No disturbances in proprioception have been described with either unilateral thalamotomy or Vim DBS. However, changes in gait and balance have been reported with bilateral DBS (Earhart et al., 2009).

Clinically, it is difficult to quantify proprioception with standard bedside techniques. The tests used lack sensitivity, reliability and are prone to rater subjectivity (Lincoln et al., 1991). Recent work from our group has utilized robotics to generate objective, reliable and highly sensitive measures to quantify position sense (Dukelow et al., 2010, 2012; Scott and Dukelow, 2011; Fung et al., 2014; Herter et al., 2014) and kinaesthesia (Semrau et al., 2013). Therefore, we evaluated the effects of Vim DBS implantation and subsequent stimulation on proprioceptive processing. We hypothesized that individuals with tremor (ET or PD) did not possess inherent impairments in kinaesthetic function, but that implantation of a DBS electrode and stimulation may disrupt kinaesthetic processing.

## Materials and methods

### Subject groups

We evaluated sensory and motor behavior in two subject groups undergoing thalamic DBS for tremor: a Surgery group [*n* = 6, (*n* = 4, ET; *n* = 2, PD)] and a Stim group [*n* = 11, (all ET)]. Subjects were recruited at Foothills Hospital in Calgary. Candidate subjects were scheduled to receive a DBS implant (Surgery group), or had an existing, programmed DBS implant (Stim group). Subjects with upper body/musculoskeletal injuries, other neurological conditions, and visual field deficits were excluded. We recruited age, sex and handedness matched control subjects. Handedness was determined via self-report. The study was approved by the University of Calgary Ethics Committee and subjects provided informed consent. Subject demographics are reported in Table 1.

**Table 1:** **Subject Demographics**.

	**Controls (Surgery) (*N* = 6)**	**Surgery group (*N* = 6)**	**Controls (Stim) (*N* = 11)**	**Stim group (*N* = 11)**	
				**ST stim (<2 years, *N* = 6)**	**LT stim (>3 years, *N* = 5)**
Age (years)	69.5 ± 3.9	69.0 ± 3.1	68.0 ± 9.5	65 ± 7.2	70 ± 6.8
Sex	6 M	6 M	8 M/3 F	5 M/1 F	3 M/2 F
Handedness	5 R/1 L	5 R/1 L	8 R/1 L	5 R/1 L	3 R/2 L
Affected side	–	5 R/1 L	–	5 R/1 L	3 R/2 L
Diagnosis	–	4 ET/2 PD	–	6 ET	5 ET
Disease duration (years)	–	17.2 ± 9.5	–	30.7 ± 17.0	24.2 ± 10.4
Stim duration (years)	–	–	–	0.8 ± 0.6	6.2 ± 2.8
CRST	–	45.5 ± 16.1 (pre-op) [*n* = 4]	–	42.6 ± 17.3 (stim OFF) [*n* = 5]	54.6 ± 17.1 (stim OFF) [*n* = 5]
		57.7 ± 11.0 (6 mo. post-op) [*n* = 3]		24.3 ± 9.8^*^ (stim ON) [*n* = 4]	30.8 ± 15.8^*^ (stim ON) [*n* = 5]

Abbreviations: ET, essential tremor; F, Female; L, Left; M, Male; PD, Parkinson Disease; R, Right; CRST, Fahn-Tolosa-Marin Clinical Rating Scale for Tremor.

* *indicates significantly different from within group stim OFF, *p* < 0.05 (paired t-test). CRST was not available in all subjects*.

### Experiment 1—surgery group

Surgery group subjects (*n* = 6) were recruited prior to unilateral DBS electrode implantation. Sensory and motor performance was evaluated robotically (described below) at two time points, prior to DBS electrode implantation [Pre-Surg, 13 ± 16.12 days prior (mean ± SD)], and immediately after implantation of the DBS electrodes, before DBS was turned on (Post-Surg, 3 ± 3.92 days).

### Experiment 2—stim group

Subjects in the Stim group (*n* = 11) had pre-existing unilateral Vim DBS stimulators to relieve tremor. Sensory and motor performance was assessed at a single time-point, at least 6 months after DBS stimulation had begun. Subjects were tested in two conditions: (1) stimulator ON and (2) stimulator OFF. Between testing conditions, subjects rested for a period of at least 20 min to ensure no lasting effects of the previous session condition. The testing order (ON/OFF or OFF/ON) was randomized to account for effects of stimulator state order. There were no aftereffects of stimulation, such as rebound tremor in this patient cohort, therefore 20 min between tests was felt to be optimal and similar to other studies (Flament et al., 2002; Ushe et al., 2004, 2006; Earhart et al., 2007, 2009; Fasano et al., 2012). Stimulation was performed with the individual's usual stimulation settings, set to be best for tremor suppression with fewest side-effects. Three subjects were included in both Experiment 1 and 2. These subjects were first evaluated in the Surgery group and were retested after receiving at least 6 months of programmed DBS stimulation.

To compare the effects of short-term and long-term stimulation, we sub-divided the Stim group into those that had received stimulation for at least 6 months, but less than 2 years [Short-term stim (ST-stim), *n* = 6], and those that had received stimulation for greater than 3 years [Long-term stim (LT-stim), *n* = 5]. This is similar to previous studies that have classified long-term stimulation as three or more years of DBS (Funkiewiez et al., 2004). Subjects in both groups actively used their stimulators for at least 12 h a day. There were no significant differences in reported disease duration between subjects in the Surgery group and the Stim group, or between subjects in the long-term (LT) and short-term (ST) stim groups (unpaired *t*-tests, *p* > 0.05).

### Robotic tasks

For all subjects in Experiment 1 and 2, proprioceptive and motor function was evaluated using the KINARM robotic exoskeleton (BKIN technologies, Kingston, ON, Canada) (Scott, 1999; Dukelow et al., 2010; Scott and Dukelow, 2011; Semrau et al., 2013). Subjects performed three tasks designed to quantify motor and proprioceptive function: (1) Visually Guided Reaching (Coderre et al., 2010), (2) Position Matching (Dukelow et al., 2010), (3) Kinaesthetic Matching (Semrau et al., 2013). Subjects sat in the wheelchair base of the robotic exoskeleton with their arms supported against gravity by arm troughs attached to the exoskeleton, and performed the three tasks in the horizontal plane of movement. For the visually guided reaching tasks, a video display was reflected onto a horizontally mounted mirror to create a virtual reality environment within the same plane as the arms and hands. For the position matching and kinaesthetic matching tasks, vision was occluded.

### Task 1: visually guided reaching

**Figure 1:**
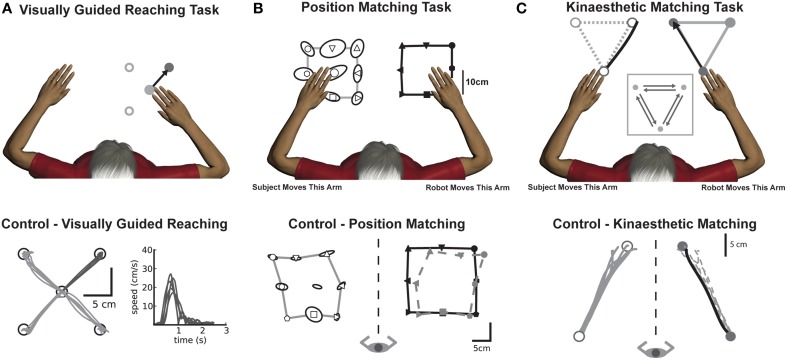
**Experimental tasks (top panels) with corresponding exemplar control data (bottom panels)**. **(A)** Visually guided reaching: Motor control was measured by quantifying reaching behavior in a standard center-out reaching task. An exemplar control (bottom panel) depicts intact reaching behavior with straight, accurate reaches to each of the four targets along with consistent hand speed performance taken from a single target. **(B)** Position matching: The robot moved the subjects' arm (top panel, right arm in example) to one of nine locations in the workspace. Subjects then mirror-matched the location of the robot movement (top panel, left arm) to where they had sensed their arm had been moved. The exemplar control (bottom panel), demonstrates that the subject (open symbols) could accurately match the locations of the robot movement (black closed symbols). The dotted gray line shows the outline of the subject's data from the outer 8 points mirrored directly onto the robot workspace for visualization purposes. **(C)** Kinaesthetic matching: The robot moved the subjects' arm (top panel, right arm in example) to one of three locations in the workspace. As soon as the subject felt the robot move their arm, they were to mirror-match the speed, direction and length of the robot-generated movement (top panel). The exemplar control (bottom panel), demonstrates that for a single direction of the task, the subject (open symbols, gray lines) was able to consistently and accurately match the direction and length of the robot-generated movement (closed symbols, black line). The dotted gray line shows subject performance mirrored directly onto the robot movement for visualization purposes.

Motor performance was quantified using a standard four-target center-out reaching task similar to that described in Coderre et al. (2010) (Figure 1A). Subjects were instructed to hold a white dot (representing their fingertip) in a red circle (center target, randomized hold time of 750–1750 ms) and wait for a peripheral target to appear. Subjects were instructed to reach as quickly and accurately as possible to one of four peripheral targets presented in a random order (Figure 1A). Subjects performed 5 reaching movements to each target. One subject in the Stim group performed an older version of the task involving 8 targets, with 8 movements in each direction for a total 64 movements. For subjects in the Surgery and Stim groups, we tested the arm contralateral to DBS implant (or planned implant). For control subjects, data from both arms were included.

### Task 2: position matching

Sense of limb position was evaluated using a previously described position matching task (Dukelow et al., 2010, 2012; Fung et al., 2014; Herter et al., 2014) that is similar to other methods (Goble et al., 2006). With vision occluded, the robot moved the subjects' arm (passive arm) to one of nine locations on one side of the workspace. Subjects were instructed to mirror-match the location that the robot had moved their arm with their opposite arm (active arm) when the robot stopped moving (Figure 1B). Each of the nine locations was pseudorandomly presented within a block. Subjects performed 6 blocks for a total of 54 movements. In all subjects with tremor, the robot passively moved the arm more affected by tremor, testing position sense in the arm affected by the tremor. Control subjects were tested on both arms. Due to technical issues in data collection for this task, we analyzed data from four subjects in the Surgery group pre-operatively and 2 post-operatively. Data were available for 8 subjects in the Stim group.

### Task 3: kinaesthetic matching

Subjects' sense of limb movement was evaluated with a kinaesthetic matching task (Figure 1C) (Semrau et al., 2013). Before each trial started, the robot moved the subjects' passive arm to one of three target locations in the workspace. After a random time delay (1500 ± 500 ms), the robot began the trial by moving the passive arm to one of the other targets at a preset speed. From target-to-target, the robot moved 20 cm with a bell-shaped velocity profile (peak speed of 0.28 m/s). As soon as subjects felt movement of the passive arm, they were to mirror-match the speed, direction and magnitude of the movement with the opposite arm (active arm). In all patients, we tested the perception of movement in the arm opposite the side of the DBS implant (the passive arm) and they mirror-matched with their unaffected arm (active arm). Control subjects were tested on both arms.

### Data analysis

#### Task 1: visually guided reaching

Data were analyzed with metrics used to quantify goal-directed reaching (Coderre et al., 2010; Debert et al., 2012; Dukelow et al., 2012) as well as metrics developed specifically to look at tremor. Briefly, we measured Postural Speed (PS), speed of the hand during rest phases of movement; Reaching Initial Direction Error (R-IDE), the amount of angular displacement during initial phase of movement; Reaction Time (RT), time from peripheral target appearance to movement initiation; Movement Time (MT), total time for subjects to complete the movement; and Max Speed (MS), peak hand speed of the movement. Additionally, we measured the effect of tremor on corrective distance after initial phase of movement (Corrective Path Length, CPL).

#### Task 2: position matching

The metrics used to quantify position matching behavior have been previously described (Dukelow et al., 2010, 2012; Debert et al., 2012). Briefly, three measures summarized position sense: Variability (Var) in end point location; Systematic shifts (Shift) of the active hand relative to mirrored position of the passive hand (translation of workspace); and Contraction/Expansion Ratio (C/E), which quantified the ratio of area matched by the active hand relative to area of the passive hand.

#### Task 3: kinaesthetic matching

Kinaesthetic behavior was quantified with several parameters that captured both spatial and temporal components of movement perception (Semrau et al., 2013). Response Latency (RL) was computed as the difference in time from robotic movement initiation in the passive arm and movement onset of the active arm. Peak Speed Ratio (PSR) was computed as the ratio between the maximum hand speed of the active and passive arms. A ratio of 1 indicated perfect matching, with a ratio of <1 indicating the active arm moved slower than the passive arm. Kinaesthetic Initial Direction Error (K-IDE) quantified sense of direction by measuring absolute angular deviation at peak hand speed between the active and passive arms. Path Length Ratio (PLR) quantified subjects' ability to match the distance of the passive movement, and was calculated by dividing total movement length of the subject's active arm by the length moved by the passive arm. A ratio of 1 indicated perfect matching, with a ratio of <1 indicating the active arm moved short than the passive arm.

#### Statistical analyses

We computed significance with One-Way ANOVAs for all results in Experiment 1, and mixed-effects ANOVAs for results in Experiment 2. All *post-hoc* comparisons were computed using Tukey tests with alpha at 0.05. Additional correlations were computed between duration of stimulation and individual robotic parameters. Data shown are mean and standard error of the mean (SEM) unless otherwise stated.

## Results

### Experiment 1—effects of vim DBS implantation surgery on sensorimotor function

#### Motor behavior: visually guided reaching

**Figure 2:**
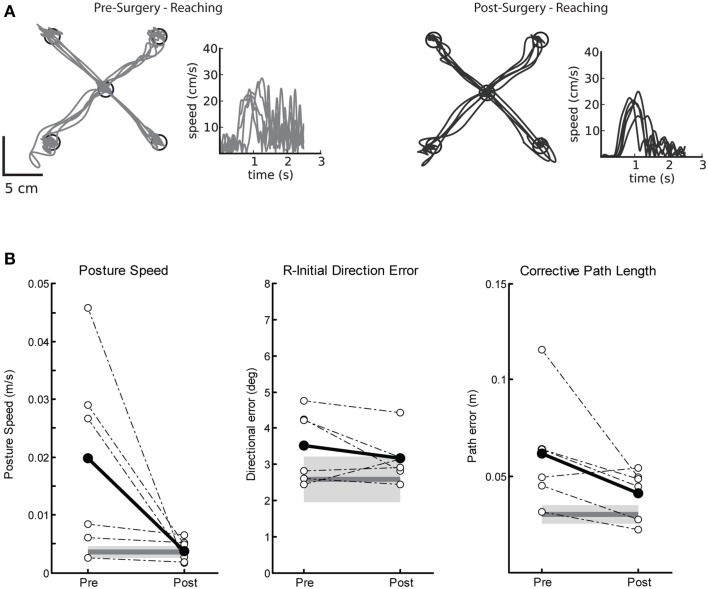
**Exemplar reaching data for a subject with tremor before and after Vim DBS implantation**. **(A)** Pre-surgery (left panel), and post-surgery reaching behavior (right panel). **(B)** Performance on three measures of reaching behavior for posture speed (left panel), a measure of resting hand speed before movement initiation; initial direction error (middle panel), a measure of directional error in the beginning phase of movement to the target; corrective path length (right panel), a measure of corrective distance covered to stabilize at the target after the initial phase of movement. Average control performance (*n* = 6) is displayed as a solid dark gray line with a gray box representing control subject variability (Standard Deviation). Average patient subject data is plotted as a solid black line, and individual subject data is plotted as dotted lines for Pre- and Post-Surgery (*n* = 6). We observed that posture speed and corrective path length improved post-surgery.

Pre-surgery impairments on the visually guided reaching task were marked by poor postural control and large corrective path lengths. Figure 2A demonstrates a single subjects' performance on the visually guided reaching task before (left panel) and after (right) Vim DBS implantation. Prior to surgery (Figure 2A, left), this subject demonstrated many corrective movements to reach the target, as well as a hand speed profile with large magnitude oscillations, characteristic of tremor. After surgery (Figure 2A, right), there were fewer corrective movements and amplitude reduction for these movements, as one would expect with reduction of tremor. Prior to DBS implantation, we observed significantly elevated posture speed (Figure 2B, left) and corrective path length (Figure 2B, right) compared to controls. After DBS implantation, we observed a significant reduction in posture speed and corrective path length, suggesting an overall improvement in motor performance after Vim DBS implantation (Table 2). We did not observe any significant differences in initial direction error (Figure 2B, middle), movement time or maximum speed (Table 2) prior to or after surgery compared to controls.

**Table 2:** **Task Performance**.

	**Controls (Surgery)**	**Surgery group**		**Controls (Stim)**	**Stim group**			
		**Pre-surgery**	**Post-surgery**		**Short-term stim**		**Long-term stim**	
					**OFF**	**ON**	**OFF**	**ON**
**VGR**								
PS (m/s)	0.004 ± 0.0003	0.02 ± 0.007^*^	0.004 ± 0.0008^**^	0.003 ± 0.0005	0.013 ± 0.007	0.003 ± 0.0006	0.03 ± 0.01^*^†‡	0.015 ± 0.005^*^†‡
R-IDE (°)	2.58 ± 0.18	3.51 ± 0.41	3.16 ± 0.28	2.50 ± 0.24	2.64 ± 0.31	2.65 ± 0.40	5.01 ± 0.95^*^†‡	5.24 ± 1.77^*^†‡
RT (s)	0.35 ± 0.02	0.41 ± 0.04	0.39 ± 0.03	0.34 ± 0.01	0.37 ± 0.02	0.35 ± 0.02	0.52 ± 0.07	0.47 ± 0.02
MT (s)	0.97 ± 0.05	0.84 ± 0.09	1.17 ± 0.16	1.12 ± 0.05	0.94 ± 0.11	1.06 ± 0.07	0.98 ± 0.18	0.98 ± 0.16
MS (m/s)	0.28 ± 0.02	0.25 ± 0.03	0.24 ± 0.04	0.24 ± 0.01	0.25 ± 0.03	0.23 ± 0.03	0.25 ± 0.04	0.27 ± 0.04
CPL (m)	0.03 ± 0.001	0.06 ± 0.01^*^	0.04 ± 0.005	0.03 ± 0.002	0.07 ± 0.03	0.04 ± 0.006	0.09 ± 0.02^*^†‡	0.09 ± 0.03^*^†‡
**PM**								
SHIFT (cm)	3.26 ± 0.54	6.21 ± 1.34	7.80 ± 0.40^*^	4.43 ± 0.78	4.84 ± 1.07	3.92 ± 0.77	7.60 ± 1.71	5.80 ± 1.97
C/E	0.66 ± 0.06	0.88 ± 0.10	0.69 ± 0.09	0.71 ± 0.07	0.75 ± 0.06	0.72 ± 0.06	0.54 ± 0.11^*^†‡	0.48 ± 0.08^*^†‡
VAR (cm)	3.99 ± 0.25	5.41 ± 0.88	3.33 ± 0.22	3.81 ± 0.30	3.49 ± 0.28	3.92 ± 0.28	5.46 ± 0.52^*^†‡	5.90 ± 1.62^*^†‡
**KIN**								
RL (ms)	493.30 ± 55.56	596.90 ± 123.77	520.77 ± 68.32	416.35 ± 66.78	309.94 ± 30.15	429.33 ± 86.22	423.49 ± 87.06	339.25 ± 40.57
PSR	1.09 ± 0.03	0.98 ± 0.07	1.05 ± 0.07	1.05 ± 0.04	1.10 ± 0.06	1.04 ± 0.05	0.83 ± 0.12^*^†‡	0.89 ± 0.10^*^†‡
K-IDE (°)	16.92 ± 1.27	17.53 ± 1.61	15.93 ± 2.17	15.72 ± 1.41	14.14 ± 1.47	14.91 ± 1.55	40.93 ± 13.93^*^†‡	34.26 ± 7.73^*^†‡
PLR	1.01 ± 0.02	1.00 ± 0.06	1.04 ± 0.05	1.04 ± 0.03	1.06 ± 0.07	1.02 ± 0.03	0.96 ± 0.10	1.07 ± 0.10

*Abbreviations: VGR, Visually Guided Reaching; PM, Position Matching; KIN, Kinaesthesia; PS, Posture Speed; R-IDE, Reaching Initial Direction Error; RT, Reaction Time; MT, Movement Time; MS, Movement Speed; CPL, Corrective Path Length; SHIFT, Systematic Shift; C/E, Contraction/Expansion; VAR, Variability; RL, Response Latency; PSR, Peak Speed Ratio; K-IDE, Kinaesthesia Initial Direction Error; PLR, Path Length Ratio. All values are mean ± SEM*.

* indicates significantly different from control

** indicates significantly different from pre-surgery

† indicates significantly different from ST ON

‡ indicates significantly different from ST OFF.

#### Proprioceptive behavior: position matching

**Figure 3:**
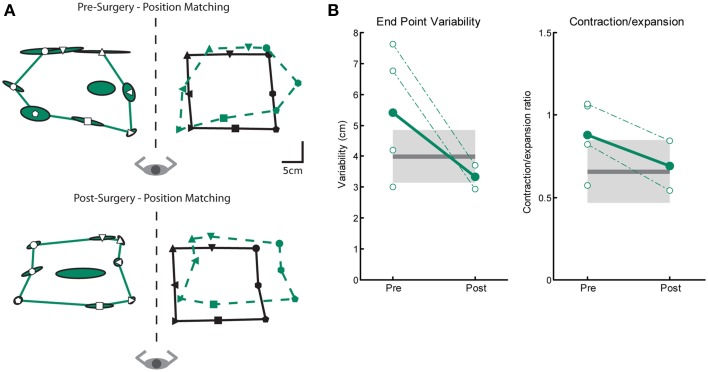
**Exemplar position matching for a subject before and after left Vim DBS implantation**. **(A)** Pre-surgery position matching behavior (top panel), and post-surgery position matching behavior (bottom panel). **(B)** Performance on two measures of position matching for pre-surgery (*n* = 4) and post-surgery subjects (*n* = 2): end point variability (left panel), a measure of subject ability to consistently match location of robot movement; workspace contraction/expansion (right panel), a measure of subject ability to conserve the shape of the workspace generated by the robot movements. Control data is indicated by the black line (mean) and gray box (SD). Average patient data is indicated by filled circles and solid line, individual patient data is indicated by open circles and dotted lines. We observed no significant differences in position matching behavior pre- or post-surgery.

We measured three parameters of position sense prior to and after Vim DBS implantation (Var, C/E and Shift). Figure 3A depicts an ET subjects' performance on the position matching task before (top) and after (bottom) DBS implantation. This subjects' performance resembles the exemplar control subject (Figure 3B) and closely matches the position of target locations demonstrating intact sense of position. We observed no difference between control subjects and tremor subjects either pre- and post-surgery in position sense for measures of variability or contraction/expansion (Figure 3B, Table 2). However, systematic shift post-surgery in 2 subjects was larger than controls (Table 2).

#### Proprioceptive behavior: kinaesthetic matching

**Figure 4:**
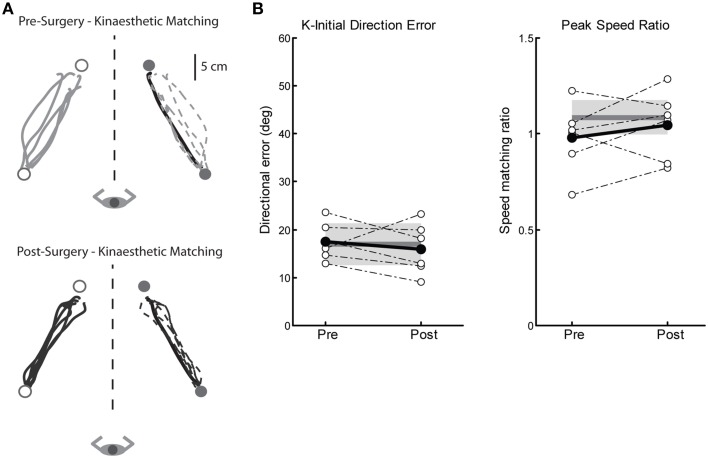
**Exemplar data for the kinaesthetic matching task for a subject before and after left Vim DBS implantation**. **(A)** Pre-surgery kinaesthetic matching behavior (top panel), and post-surgery kinaesthetic matching behavior (bottom panel). **(B)** Performance on two measures of the kinaesthetic matching task show no impairment in sense of movement direction (initial direction error, left panel) or ability to match the speed of the robot-generated movement (peak speed ratio, right panel). Average control performance (*n* = 6) is displayed as a solid dark gray line with a gray box representing control subject variability (Standard Deviation). Average patient subject data is presented as a solid black line, with individual subjects as dotted lines. Overall, we observed intact kinaesthetic behavior both pre- (*n* = 6) and post-surgery (*n* = 6).

We measured four parameters of kinaesthesia before and after Vim DBS implantation (K-IDE, PSR, RL, and PLR). Figure 4A depicts a tremor subjects' kinaesthetic behavior before (top) and after surgery (bottom). This subjects' performance closely mimics that of our exemplar control (Figure 1C), by accurately matching both direction and magnitude of the robotically moved limb. On average, subjects in the Surgery group demonstrated no kinaesthetic impairments before or after surgery, compared to control subjects across all four parameters (Figure 4B, Table 2).

### Experiment 2—effects of long-term stimulation on sensorimotor function

#### Motor behavior: visually guided reaching

**Figure 5:**
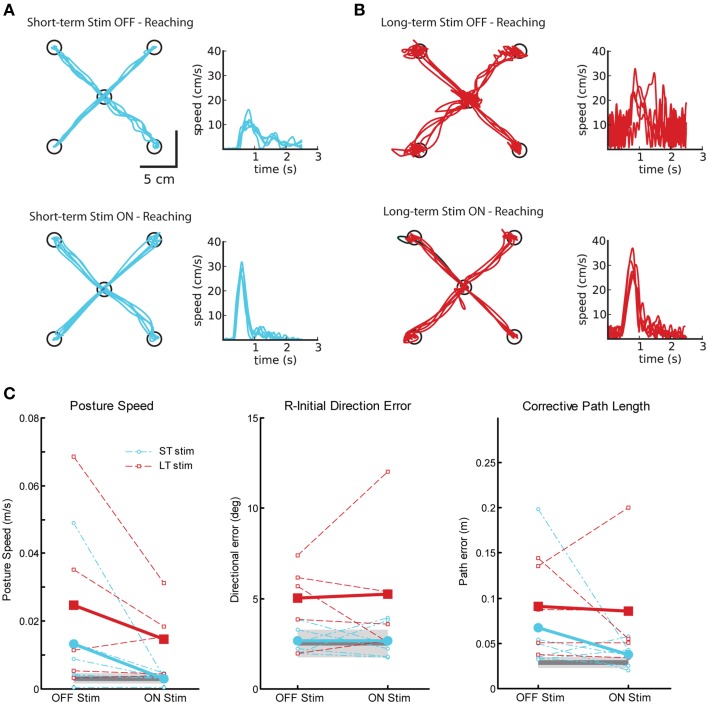
**Exemplar reaching data for subjects OFF and ON Vim DBS**. **(A)** Reaching behavior for a single subject from the short-term stim group OFF (top panel) and ON stimulation (bottom panel). **(B)** Reaching behavior for a single subject from the long-term stim group OFF (top panel) and ON stimulation (bottom panel). **(C)** Performance on three measures of reaching behavior: posture speed (left panel), a measure of resting hand speed before movement initiation; initial direction error (middle panel), a measure of directional error in the beginning phase of movement to the target; corrective path length (right panel), a measure of corrective distance covered to stabilize at the target after the initial phase of movement. Average control performance (*n* = 11) is displayed as a solid dark gray line with a gray box representing control subject variability (Standard Deviation). Average patient subject data is presented as thicker solid lines (ST, blue; LT, red), with individual subject data as thinner dotted lines. Subjects in both ST (*n* = 6) and LT (*n* = 5) groups show improvement in posture speed with stimulators ON.

Figure 5 displays reaching behavior for two exemplar subjects with tremor, one from the ST-stim group (Figure 5A) and one from the LT-stim group (Figure 5B) for testing OFF and ON stimulation. In both, subjects we were able to qualitatively identify a reduction in tremor from OFF (Figures 5A,B, top) to ON stimulation (Figures 5A,B, bottom) in both the hand path and speed profiles, consistent with Clinical Rating Scale for Tremor (CRST) scores (Table 1). As expect, DBS ON state significantly reduced posture speed (i.e., postural tremor) in both the ST and LT-stim groups (Figure 5C, left, Table 2). The LT-stim group had a significantly larger R-IDE than either the control group or the ST-stim group (Figure 5C, middle, Table 2). Additionally, there was a non-significant decrease in CPL when subjects were tested with the stimulator ON. In the ON condition, there was significantly larger path length found in the LT-stim group compared to ST-stim and control groups (Figure 5C, right, Table 2). For timing and speed-based parameters, we observed a significantly longer RT for the LT-stim group, but did not observe any other differences for parameters of movement time or max speed across groups or conditions (Table 2).

#### Proprioceptive behavior: position matching

**Figure 6:**
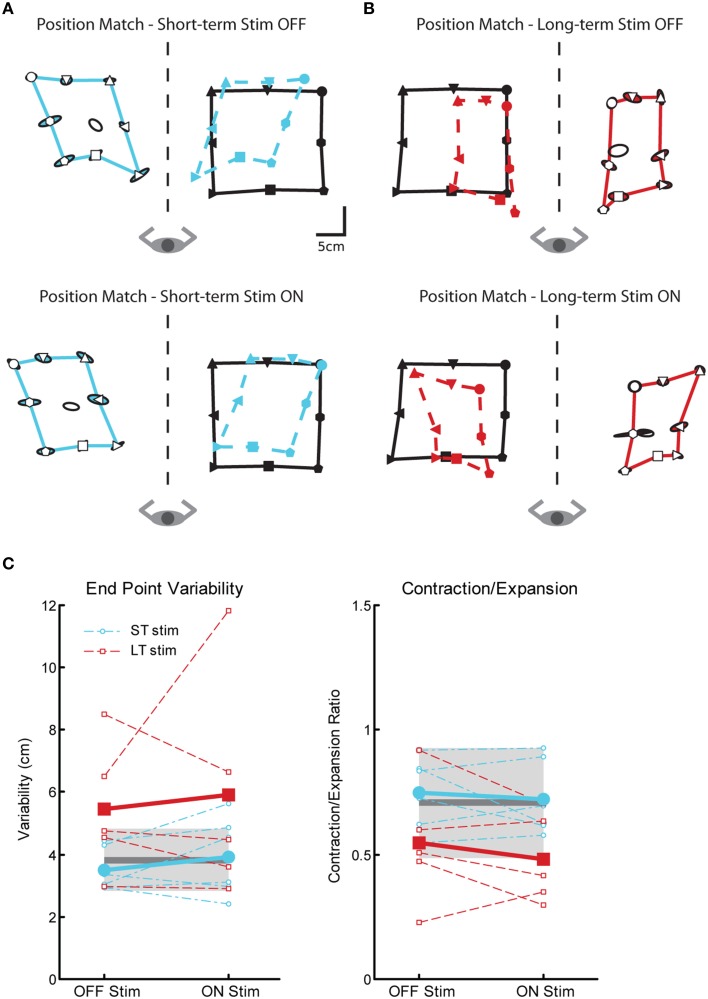
**Exemplar position matching for a short-term stim subject with a left Vim DBS implant (A) and a long-term stim subject with a right Vim DBS implant (B), OFF (top panels) and ON stimulation (bottom panels). (C)** Performance on two measures of position matching: end point variability (top panel), a measure of ability to consistently match location of the robot movement; workspace contraction/expansion (bottom panel), a measure of subject ability to conserve the shape of the workspace generated by the robot movements. Average control performance (*n* = 11) is displayed as a solid dark gray line with a gray box representing control subject variability (Standard Deviation). Average patient subject performance is displayed as a thick line, with thinner dotted lines representing individual subjects. Subjects in the LT-stim (red, *n* = 5) group displayed increased variability and a tendency to contract the workspace compared to controls and the ST-stim group (blue, *n* = 6).

We observed significant impairments in position sense for subjects in the LT-stim group compared to controls and subjects in the ST-stim group, regardless if stimulation was OFF or ON. In Figures 6A,B, the subject in the ST-stim group performed normally ON and OFF stimulation. However, the subject in the LT-stim group showed workspace contraction while stimulation was ON (Figure 6B, bottom) and workspace contraction and shift (leftward) when stimulation was OFF (Figure 6B, top). We observed significant impairments in variability for the LT-stim group compared to both controls and the ST-stim group (Figure 6C, left, Table 2). Additionally, subjects in the LT-stim group contracted the workspace, essentially reducing the area of the matched targets. The LT-stim group was significantly impaired compared to both controls and the ST-stim group for measures of C/E, regardless of stimulation state (Figure 6C, right, Table 2). For measures of shift, we failed to observe any significant differences across condition or group (Table 2).

#### Proprioceptive behavior: kinaesthetic matching

**Figure 7:**
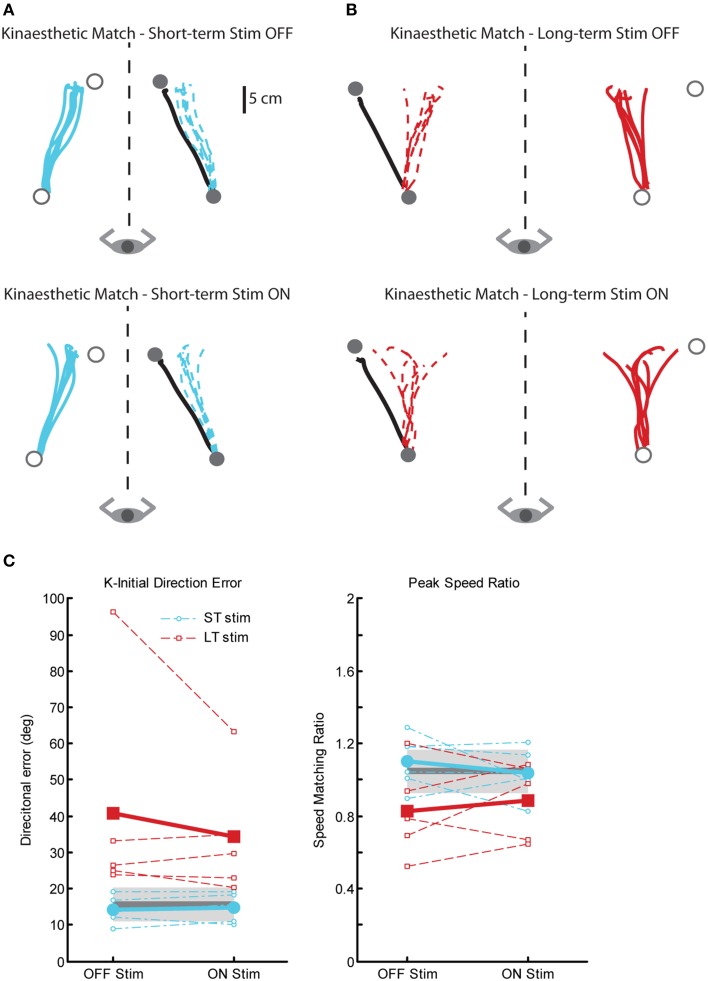
**Exemplar data for the kinaesthetic matching task in a ST-stim subject with left Vim DBS (A) and a LT-stim subject with right Vim DBS (B), OFF (top panels) and ON stimulation (bottom panels). (C)** Performance on two measures of the kinaesthetic matching task show that all subjects in the LT-stim group (*n* = 5) make large directional error (K-IDE, left panel), and have difficulty modulating their hand speed to match the speed of the robotic movement, with a tendency to move more slowly (PSR, right panel) compared to controls (*n* = 11) and ST-stim subjects (*n* = 6). Average control performance (*n* = 11) is displayed as a solid dark gray line with a gray box representing subject variability (Standard Deviation). Average patient subject data is displayed as a thicker line, with thinner dotted lines representing individual subjects.

There were also significant impairments in kinaesthetic matching for the LT-stim group compared to both ST-stim and control groups, regardless of stimulation state. Figure 7 demonstrates exemplar kinaesthetic matching for a subject in the ST-stim group (Figure 7A) and the LT-stim group (Figure 7B). The ST-stim subject performs well by matching both direction and path length of the robot movement accurately both OFF and ON stimulation (Figure 7A, top and bottom, respectively), and closely resembles the exemplar control in Figure 1C. The LT-stim subject performs poorly both OFF and ON stimulation (Figure 7B, top and bottom, respectively), by failing to accurately match direction and amplitude of the robot movement. The LT-stim group made significantly larger errors of K-IDE compared to control and ST-stim groups, regardless if they were ON or OFF stimulation (Figure 7C, left, Table 2). Additionally, subjects in the LT-stim group had significant difficulty accurately matching and tended to underestimate speed of the robot movement compared to control and ST-stim groups, regardless of stimulation state (Figure 7C, right, Table 2). There were no significant differences in RL or PLR (Table 2).

#### Relationship of stimulation duration and performance on robotic parameters

To supplement our findings, we correlated each of the robotic parameters with duration of stimulation (in years) without consideration of classification to the ST-stim or LT-stim groups. Similar to the results described above (Table 2), some parameters of the reaching task (PS, R-IDE, CPL, RT) showed significant effects of duration (Table S1). Additionally, we observed significant relationships between duration of stimulation and impairment on several parameters of PM and KIN both ON (C/E, K-IDE) and OFF stimulation (PSR) (Table S1), in agreement with results from the mixed-effects ANOVA (Table 2).

## Discussion

Surgical implantation of DBS electrodes improved motor control in patients with tremor, while causing no change in proprioception. Additionally, no proprioceptive deficits were observed prior to implantation, suggesting that proprioceptive deficits are not inherent to individuals with tremor. Proprioceptive impairments appeared only in those only subjects using Vim DBS for greater than 3 years, regardless of stimulators being turned ON or OFF at the time of testing, suggesting that long-term Vim stimulation may disrupt proprioceptive processing.

### Immediate effects of surgery

Consistent with clinical experience, we observed that subjects in the Surgery group had significant motor impairments that subsided after implantation of DBS in the Vim nucleus of the thalamus (Figure 2). The improvements in motor function seen immediately after surgery are thought to be due to the microlesion (microthalamotomy) effect that occurs temporarily as a result of electrode implantation (Sitburana et al., 2010). In contrast, upon evaluating proprioception, we observed that when subjects were tested pre- and post-surgery, they displayed no inherent proprioceptive impairments or proprioceptive deficits as a result of surgery. Overall, pre-surgery and post-surgery behavior for the position sense task was relatively normal. However, we did see a small, but statistically significant increase during post-surgery testing in the shift parameter. This finding should be interpreted with caution as we have observed neurologically intact adults with up to 10 cm of systematic shift in previous studies (Dukelow et al., 2010, 2012; Debert et al., 2012; Herter et al., 2014).

For the kinaesthetic matching task, the pre- and post-surgery performance of subjects with tremor closely mirrored performance of controls. Of the two modalities of proprioception measured in this study, we predicted it was more likely that kinaesthesia would be negatively impacted by surgical electrode implantation in Vim. Vim is the “kinaesthetic nucleus” and cells respond to movement about joints reflecting “sensation of movement” (Hirai et al., 1989; Ohye et al., 1989), while position sense is thought to be represented in ventral caudal thalamus (Vc) (Mountcastle, 1958; Hassler, 1959; Narabayashi, 1989), adjacent to Vim. However, we observed that implantation of the DBS electrode in the Vim nucleus did not produce significant deficits in either position sense or kinaesthesia.

### Effects of stimulation over time

Qualitatively, Vim stimulation improved motor performance on the visually guided reaching task (Figures 5A,B). Surprisingly, arm kinematics have been used in only a few studies to quantify the severity of tremor or tremor improvement with stimulation (Deuschl et al., 2000; Beuter et al., 2001; Flament et al., 2002; Chen et al., 2006; Herzog et al., 2007; Khandwala et al., 2009; Groppa et al., 2014). Importantly, tremor in both the ST-stim and LT-stim groups decreased when stimulation was turned ON as measured by posture speed and corrective path length parameters. Previous studies have noted impairments in gait and postural stability as a result of stimulation (Pinter et al., 1999; Earhart et al., 2009; Fasano et al., 2012), but the impact of long-term stimulation on upper limb kinematics has not been investigated.

Somewhat surprisingly, we observed a small, but statistically significant difference in the magnitude of R-IDE for the LT-stim group compared to the ST-stim group and controls in visually guided reaching, both ON and OFF stimulation. However, the magnitude of this difference was very small (~2°). This suggests that long-term stimulation may negatively impact motor control very slightly. Alternatively, it is possible that the benefits of stimulation may gradually dissipate over time with long-term usage (Koller et al., 2001) or that progression of tremor may play a role (Favilla et al., 2012). Long-term DBS was also associated with significantly longer reaction times compared to both controls and short-term DBS subjects. However, the magnitude of this difference was small and is similar to reaction times previously reported for visually-evoked motor behavior in essential tremor (Flament et al., 2002). Despite these slight differences in R-IDE and RT, the ST and LT-stim groups were not significantly different from the control group in the maximum speed measure, suggesting that both groups were able to generate appropriately normal speeds for goal-directed reaching movements.

Subjects in the LT-stim group displayed significant impairments on the position matching task that were not observed in the ST-stim group. Regardless of whether DBS was ON or OFF, significant impairments in position sense were observed as increased endpoint variability and a tendency to contract the workspace. In the kinaesthesia task, the LT-stim subjects made directional errors that were nearly ten-fold larger (~20°) than those exhibited in the reaching task. Further, despite the fact the LT-stim group demonstrated the ability to accurately match the speed of controls in the reaching task, they were incapable of generating movement speeds comparable to controls and ST-stim subjects in the kinesthesia task. These findings suggest long-term chronic Vim DBS stimulation can negatively affect proprioception.

To further support the effect that we see of long-term Vim DBS on proprioception, we also tested a subject with 3 years of Vc DBS for neuropathic hand pain (data not included in present study), on the same protocol. She performed similarly to controls both ON and OFF stimulation for all three tasks described in this study. While we only have one subject with a non-Vim thalamic DBS system, she serves as a case control for our LT-stim group. There were no proprioceptive deficits identified despite her use of long-term DBS in an adjacent, but functionally separate thalamic nucleus. This suggests that there may be localized thalamic network plasticity induced specifically in response to long-term Vim stimulation.

### Limitations

To further support our findings, ideally we would have also conducted pre- and post-operative nerve conduction studies to determine the absence of peripheral sensory deficits. Such testing would allow us to confirm that the proprioceptive deficits that we see are completely due to long-term Vim DBS, and are not compounded by peripheral neuropathy. However, all individuals in Experiments 1 and 2 underwent bedside clinical exams that indicated no sensory dysfunction. Additionally, no subjects in either experiment complained of sensory deficits during pre- or post-operative periods. Our study examined proprioceptive sensation at the shoulder and elbow joints, which would presumably only be affected in very late stages of a length dependent neuropathy when patients are typically symptomatic from their neuropathy.

Another limitation to our study is that we did not assess a control group of non-stimulated tremor patients. Ideally, we would have tested individuals with similar duration and severity of tremor as those individuals in our long-term stimulation group to confirm that the results that we see are due to long-term Vim DBS and not disease duration or severity.

### Clinical and mechanistic implications

Vim DBS can provide a life-changing improvement in quality of life for many people with tremor. None of the subjects in our study noticed or complained of any proprioceptive deficits, nor could routine neurological exam identify such deficits. These subjects would choose to continue using their stimulator given the benefits for reducing their tremor. The proprioceptive deficits we observed are subclinical and subtle compared to other neurologically impaired populations that we have studied (Dukelow et al., 2010; Debert et al., 2012; Semrau et al., 2013), but the results are powerful in that they suggest that long-term stimulation of Vim may potentially lead to thalamic, thalamocortical, or corticothalamic connectivity changes that may impact proprioception. If the deficits that we observed were just an immediate effect of stimulation, then the impairments would have resolved when the stimulator was turned OFF. It also suggests that the prevailing opinion that DBS is entirely reversible, may not be entirely correct. While we see that proprioceptive deficits are not inherent to individuals with tremor or as a result of Vim implantation surgery (as in our Surgery group), we cannot absolutely rule out the possibility that proprioceptive deficits may develop and progress over the course of the disease (Favilla et al., 2012).

There are few studies that address the non-motor effects of Vim stimulation, such as proprioception. Postural stability and gait (Pinter et al., 1999; Earhart et al., 2009; Fasano et al., 2012) was found to be impaired both ON and OFF stimulation (Earhart et al., 2009). While essential tremor is known to result in gait ataxia as the condition progresses, most of the patients reported in that study also had stimulators active for over 3 years. It is possible that the impairments seen in gait and postural control may be exacerbated by underlying proprioceptive abnormalities as a result of long-term Vim stimulation. Additionally, little is known about the long-term effects of DBS outside of sustained functional improvements.

In summary, surgical implantation of DBS had no effect on kinaesthetic processing in tremor patients. However, long-term DBS induced deficits in kinaesthetic processing are subclinical and would not be identified during a usual sensory neurologic exam. This suggests that DBS may not always be reversible and that long-term stimulation may potentially induce plastic changes in brain networks.

### Conflict of interest statement

The authors declare that the research was conducted in the absence of any commercial or financial relationships that could be construed as a potential conflict of interest.
