# Supplementary material for: Disruption in proprioception from long-term thalamic deep brain stimulation: a pilot study
Source: Front Hum Neurosci. 2015 May 1;9:244. doi: 10.3389/fnhum.2015.00244 (PMC4416461; doi:10.3389/fnhum.2015.00244)
Supplement: Supplementary file 1 [file Table1.DOCX]

Supplemental Table 1: Correlations between robotic parameters and duration of chronic DBS

| Robotic  Task | Robotic Parameter | r-value | |  |
| --- | --- | --- | --- | --- |
|  |  | OFF stim | ON stim | |
| Visually Guided Reaching |  |  |  | |
|  | Posture Speed | 0.41 | 0.78* | |
|  | Reaching-Initial Direction Error | 0.82* | 0.56 | |
|  | Corrective Path Length | 0.38 | 0.81* | |
|  | Reaction Time | 0.66* | 0.75* | |
|  | Movement Time | -0.10 | -0.41 | |
|  | Movement Speed | 0.10 | 0.41 | |
| Position Matching |  |  |  | |
|  | Variability | 0.26 | 0.23 | |
|  | Contraction/Expansion | -0.55 | -0.74* | |
|  | Shift | 0.29 | 0.47 | |
| Kinesthetic Matching |  |  |  | |
|  | Kinesthesia-Initial Direction Error | 0.58 | 0.68* | |
|  | Peak Speed Ratio | -0.61* | -0.22 | |
|  | Response Latency | -0.02 | -0.40 | |
|  | Path Length Ratio | -0.30 | 0.40 | |

*p < 0.05
